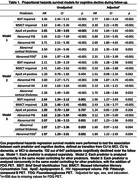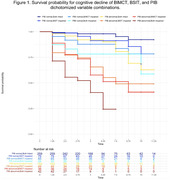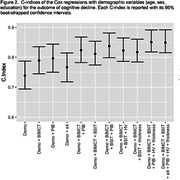# Comparison of olfactory and cognitive measures to neuroimaging biomarkers in the prediction of cognitive decline and dementia in the MCSA cohort

**DOI:** 10.1002/alz.084722

**Published:** 2025-01-09

**Authors:** Davangere Devanand, Seonjoo Lee, David S. Knopman, Maria Vassilaki, Jose A. Luchsinger, Jeffrey N Motter

**Affiliations:** ^1^ Columbia University Irving Medical Center, New York, NY USA; ^2^ New York State Psychiatric Institute, New York, NY USA; ^3^ Mayo Clinic, Rochester, MN USA; ^4^ Columbia University Medical Center, New York, NY USA

## Abstract

**Background:**

Inexpensive, non‐invasive tests may improve the identification of persons at increased risk for cognitive decline and dementia. We compared impairment in odor identification and global cognition with neuro‐imaging biomarkers to predict cognitive decline and dementia in the population‐based Mayo Clinic Study of Aging (MCSA).

**Method:**

At the 2008 assessment, 647 participants who were ≥ 55 years old with at least one follow‐up had the following procedures: modified Blessed Information‐Memory‐Concentration Test (BIMCT), 12‐item Brief Smell Identification Test (BSIT), brain magnetic resonance imaging (MRI), and Positron Emission Tomography (PET) amyloid imaging with 11C‐Pittsburgh compound B (11C‐PiB). A subsample (n=556) also had 18F‐fluorodeoxyglucose (18F‐FDG) PET scans. The utility of these baseline measures to predict cognitive decline (cognitively unimpaired to mild cognitive impairment [MCI] or dementia, or MCI to dementia) was examined during follow‐up (mean 8.1 SD 3.4 years). Transition to dementia was a secondary outcome.

**Result:**

Among 647 participants without dementia (mean age 74.0 SD 8.1 years; 54.4% male) at baseline, 102 participants manifested cognitive decline and 34 participants transitioned to dementia. In Cox regression survival analyses for cognitive decline, PiB PET showed the strongest predictive utility (hazard ratio HR 5.05, 95% CI = 3.32‐7.69, p<.001). Impaired BSIT (HR 3.46, 95% CI = 2.34‐5.11, p<.001), impaired BIMCT (HR 3.63, 95% CI 2.46‐5.36, p<.001), MRI and FDG measures were also significant predictors. After adjusting for age, sex and education, these predictors remained significant with attenuated effects (PiB HR 3.89, 95% CI 2.54‐5.95, p<.001; BSIT HR 2.77, 95% CI 1.85‐4.14, p<.001; BIMCT HR 3.3, 95% CI 2.21‐4.95, p<.001). After adding these demographic variables in the model, the combination of BSIT and BIMCT showed strong predictive utility (C‐index=0.85), similar to PiB PET (C‐index=0.84). Combining all predictors showed a C‐index of 0.89. These measures showed similar but stronger results for dementia transition.

**Conclusion:**

Tests of global cognition and odor identification provide a brief, non‐invasive, inexpensive method comparable to PET amyloid imaging for identifying community‐dwelling persons likely to decline cognitively or transition to dementia. Other potential uses for this combination include identification of persons likely to benefit from disease‐modifying medications and determination of eligibility for prevention trials.